# Subcutaneous Abatacept in New Onset Type 1 Diabetes: Clinical and Immunological Effects

**DOI:** 10.1002/dmrr.70074

**Published:** 2025-08-11

**Authors:** Samuel T. Jerram, Jennie H. M. Yang, Evangelia Williams, Clara Domingo‐Vila, Yuk‐Fun Liu, Mark Peakman, R. David Leslie, Timothy Tree

**Affiliations:** ^1^ Blizard Institute Queen Mary University of London London UK; ^2^ Wolfson Diabetes and Endocrine Clinic Addenbrooke's Hospital Cambridge University Hospitals NHS Foundation Trust Cambridge UK; ^3^ Department of Immunobiology School of Immunology and Microbial Sciences King's College London London UK; ^4^ National Institute for Health Research Biomedical Research Centre Guy's and St. Thomas' NHS Foundation Trust and Kings College London London UK; ^5^ Department of Diabetes and Endocrinology Guy's and St. Thomas' NHS Foundation Trust London UK; ^6^ Department of Diabetes School of Life Course Sciences King's College London London UK; ^7^ Institute of Diabetes Endocrinology and Obesity King's Health Partners London UK

**Keywords:** Abatacept, diabetes, immunotherapy

## Abstract

Abatacept is a CTLA4‐Ig fusion protein that blocks CD80/CD86‐dependent T‐cell co‐stimulation. When administered, Abatacept limits, to a variable degree, loss of stimulated C‐peptide secretion in patients with newly‐diagnosed type 1 diabetes (T1D), while reducing both circulating memory CD4^+^ T‐cells and T follicular helper (Tfh) cells; however, its precise mechanism of action is not known. To investigate this effect, we studied 12 patients, using multi‐parameter flow cytometry, who each self‐administered Abatacept in subcutaneous formulation for 6 months within 100 days of diagnosis. Abatacept treatment impacted the CD4^+^ T cell memory compartment, inducing a reduction in T‐effector cells across both conventional (Tconv) and regulatory (Treg) sub‐populations. A reduction in activated Tfh cells (CXCR5^+^PD1^+^ICOS^+^), previously described with intravenous therapy, was replicated and extended. An integrated baseline immunological phenotype predicted Abatacept‐induced preservation of C‐peptide.

## 1 Introduction

While insulin therapy remains the mainstay of treatment of Type 1 diabetes (T1D), there has been recent interest in immunotherapeutic approaches to halt or limit beta cell destruction [1]. Abatacept, a CTLA4‐Ig fusion protein that blocks CD80/CD86‐dependent T‐cell co‐stimulation, has been shown to limit loss of stimulated C‐peptide secretion in some patients with newly‐diagnosed T1D [2]; however, the therapeutic response is heterogeneous, and the precise mechanism of action is not known. A solution to the variability of response would be the identification of reliable biomarkers of therapeutic efficacy.

Previous analysis of patients treated with intravenous Abatacept therapy demonstrated an alteration in CD4^+^ T cell subsets, and that pre‐treatment increase in CD4^+^ central memory (Tcm) Tcells predicted a subsequent decline in C‐peptide [3]. Circulating T follicular helper (cTfh) cells markedly reduce during Abatacept therapy, with initial frequency also predicting therapeutic response [4]. We sought to design a biomarker‐rich, mechanism‐based clinical study to replicate key findings, using a self‐administered subcutaneous formulation of Abatacept, in patients aged 18–45 years, within 100 days of diagnosis of diabetes, with at least two diabetes‐related antibodies.

## 2 Methods

The Effect of Co‐stimulation Blockade on T Cell Autoreactivity in New Onset Type 1 Diabetes (COMBAT) study was conducted with full ethical approval and HRA oversight (London Fulham Research Ethics Committee ref 17/LO/0076). All patients were treated with subcutaneous Abatacept for 24 weeks, followed for a further 24 weeks off therapy, and underwent a 2‐h mixed meal tolerance test (MMTT) at baseline and at the end of the study (48 weeks). Immune cell phenotyping was undertaken by 5 panel flow cytometry on fresh peripheral blood samples pre‐treatment and at 12, 24, 36 and 48 weeks, utilising 5 panels of between 9 and 14 immune cell markers. A graphical representation of the study design is included in Figure S1. An example of gating strategy is included in Figure S2.

Data sets were first assessed for normality with the D'Agostino & Pearson Normality Test. Those that passed normality were then analysed using one‐way analysis of variance (ANOVA); those that did not were analysed using the Friedman Test.

## 3 Results

Thirteen patients were recruited to the study. One patient developed sepsis requiring hospitalisation during the treatment phase of the study (patient withdrawn). This was not identified as an adverse event related to the treatment. One patient reached the end of the study and provided samples for immunological analysis at all time‐points, but was unable to complete the end of study MMTT due to persistently elevated capillary blood glucose. Table 1 lists the baseline characteristics of the 13 patients recruited to the study.

**TABLE 1:** Baseline characteristics of study subjects and age‐matched controls.

Characteristic	Treatment group	Age‐matched controls
Number of patients	13	8
Mean age (years)	29.0	28.9
Body Mass Index (kg/m^2^)	23.3	23.1
Gender (% male)	46.2	75
Mean time from diagnosis to first sample (days)	87.8	95
Mean glycated haemoglobin (mmol/mol)	67.7	62.5

### 3.1 Subcutaneous Abatacept Reduced C‐Peptide Decline

**FIGURE 1:**
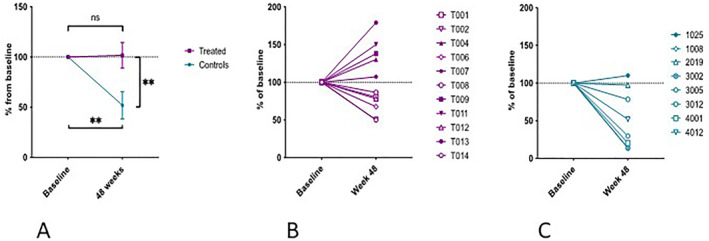
Percentage change from baseline of AUC c‐peptide/AUC glucose ratios for Abatacept‐treated patients (*n* = 11) and control patients (*n* = 8) (A) and individual values for treated patients (B) and controls (C). ** = *p* < 0.01.

The mean area under the curve (AUC) C‐peptide/AUC glucose following 2‐h Mixed Meal Tolerance Test (MMTT) did not significantly reduce at 48 weeks (*p* = 0.45). In contrast, the mean AUC c‐peptide/AUC did reduce significantly over the same timeframe in age‐matched controls (Table 1) selected from an historical study [5] (*p =* 0.0094). Within the treatment group, there was a range of responses to therapy at 48 weeks (Figure 1).

### 3.2 Abatacept Altered the Balance of CD4^+^ Naïve/Memory Subsets and Reduced Activated Follicular T Cells

**FIGURE 2:**
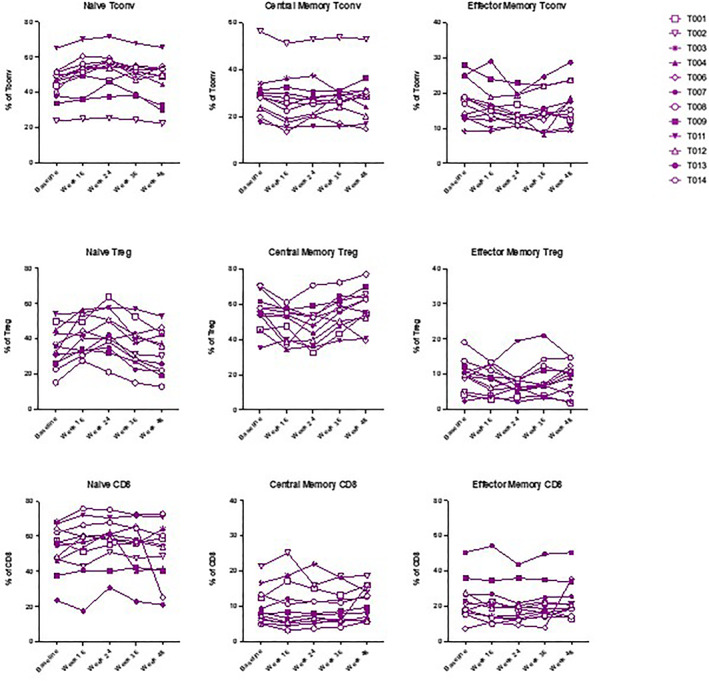
Spaghetti plots of naïve/memory cells within the Tconv, Treg and CD8 subpopulations.

**FIGURE 3:**
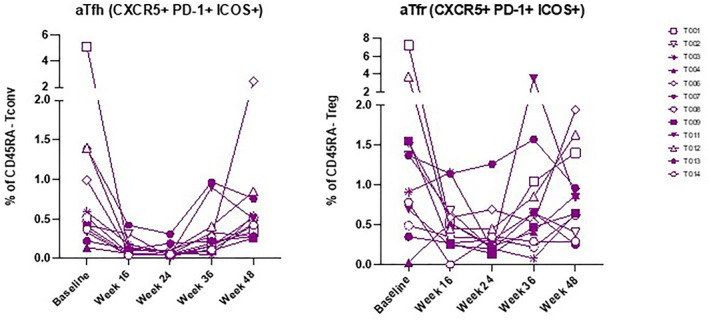
Spaghetti plots of aTfh and aTfr within Tconv and Treg subpopulations.

There was a significant increase in frequency of naïve T cells (Tn) and a significant decrease in effector memory T cells (Tem) (identified through the expression of CD45RA and CD62L) after 24 weeks of therapy, within the Tconv, Treg and CD8+ subpopulations (*p* < 0.05 throughout). There was a significant decrease in central memory T cells (Tcm) at 24 weeks within the Tconv and Treg subpopulations but not within CD8^+^ cells (*p* < 0.001 for the Tconv and Treg populations) (Figure 2). After 24 weeks of therapy, the frequency of activated circulating follicular cells, defined by the expression of CXCR5, ICOS and PD‐1, decreased in both the Tconv subpopulation (aTfh cells) (*p* < 0.0001) and Treg subpopulation (aTfr cells) (*p* = 0.0031) (Figure 3). In all instances, cell frequencies returned to baseline after a further 24 weeks of therapy.

### 3.3 Baseline Immunological Profile May Predict Response to Abatacept Therapy

**FIGURE 4:**
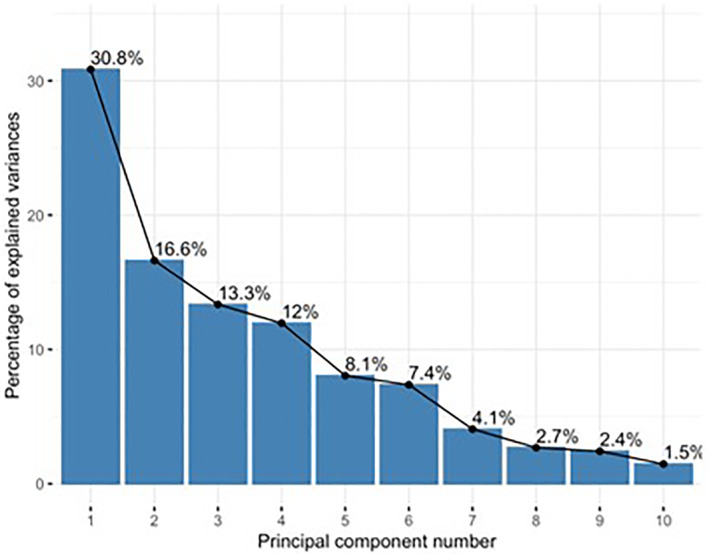
Percentage contribution of individual principal components to intergroup variance.

**FIGURE 5:**
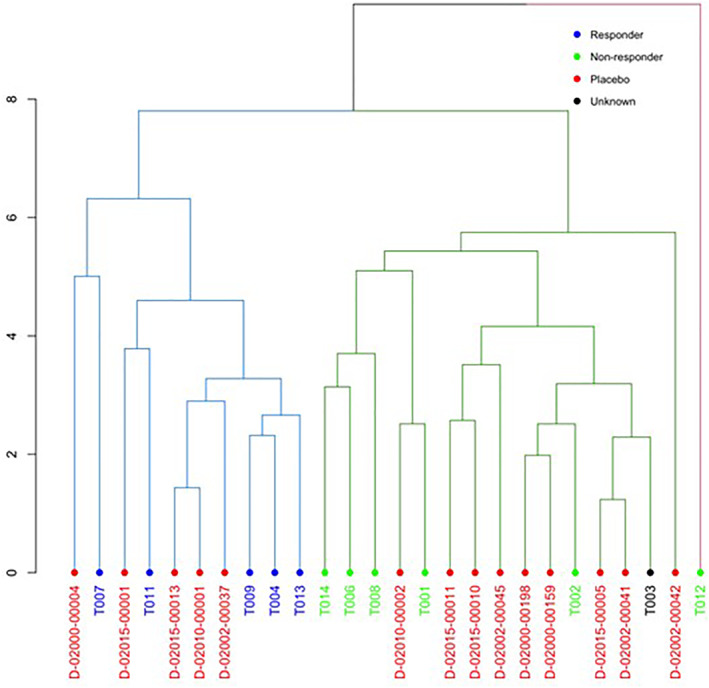
Hierarchical clustering dendrogram demonstrating separation of responders (blue) and non‐responders (green) with even distribution of controls (red).

Patients were divided into responders (*n* = 5) and non‐responders (*n* = 6) on the basis of increase or decrease in AUC C‐peptide/AUC Glucose from baseline. Baseline cell frequencies from the 11 cell populations noted to show the greatest divergence between either responders or non‐responders and controls were identified [Table 2]. Principal component analysis (PCA) was performed using data from these 11 parameters for responders, non‐responders and control subjects from the historical study described above. PCA demonstrated clear separation of the responder and non‐responder groups, with PC1 contributing 30.8%, and PC2 16.6%. Furthermore, control subjects were found to be evenly distributed between the two Abatacept groups (Figures 4 and 5).

**TABLE 2:** Eleven parameters demonstrating major variation between responders and non‐responders at baseline.

Parameter	Variation from controls	
	Responder	Non‐responder
Wbc count	Decreased	
NK count, CD56lo CD16+ NK count	Increased	
%CD69+ granulocytes	Decreased	
%CD69+ neutrophils	Decreased	
%CD69+ eosinophils	Decreased	
%CD15s+ mTeff	Decreased	
%Ki67+ mTeff	Decreased	
% Th2‐like Treg	Increased	
%Th1‐Th2‐like Treg		Decreased
%CD8+ Tscm		Increased
%aTfr		Increased

## 4 Conclusion

Subcutaneous Abatacept showed a clear clinical response comparable to that observed with the previously reported intravenous formulation.

Immunological interrogation confirmed the previously reported increase in Tn cells during treatment, now found to be due to an increase across Tconv, Treg and CD8^+^ subpopulations, with the novel observation that Tem cell frequency decreased. The previously reported reduction in aTfh cells, returning to baseline after therapy, was also replicated, with the novel finding that this involves both Tconv (aTfh) and Treg (aTfr) subpopulations. Previously, it was suggested that loss of CXCR5^+^ Tfh cells with Abatacept therapy results from loss of Tcm cells, as the majority of CXCR5^+^ cells are central memory cells. We found that Tfh cell reduction was independent of any reduction in memory T cells. This novel observation supplement evidence that aTfh cells play a central role in autoimmune diseases.

We demonstrated, through five‐panel flow cytometry, that PCA of cell populations including Tfh cell frequency could serve as a predictive model for response to Abatacept therapy.

## Author Contributions

S.J. contributed to the conception and design of the work, data curation and data cleaning, data analysis, validation, interpretation of results, drafting the article and revising the draft. J.Y., E.W. and C.D.V. contributed to data curation and cleaning, data analysis and revision of the draft. Y.F.L. contributed to conception and design of work, data curation, revision of the draft and supervision. M.P. contributed to conception and design of work, revising the draft and supervision. R.D.L. contributed to conception and design of work, drafting the article, revising draft and supervision. T.T. contributed to conception and design of work, data analysis, interpretation of results, revision of draft and supervision.

## Conflicts of Interest

The authors declare no conflicts of interest.

## Peer Review

The peer review history for this article is available at https://www.webofscience.com/api/gateway/wos/peer-review/10.1002/dmrr.70074.

## Supporting information


**Figure S1:** Study design. Graphical representation of study design showing timing of treatments and evaluation of immunological and metabolic measurements. Visits V2‐V6 included blood draw for immune cell phenotyping. Visits V2 and V6 included MMTT.

**Figure S2:** Example flow cytometry gating strategy (Treg panel).

## Data Availability

The data that support the findings of this study are available from the corresponding author upon reasonable request.
