# Supplementary figures and images for: Subcutaneous Abatacept in New Onset Type 1 Diabetes: Clinical and Immunological Effects
Source: Diabetes Metab Res Rev. 2025 Aug 11;41(6):e70074. doi: 10.1002/dmrr.70074 (PMC12340168; doi:10.1002/dmrr.70074)

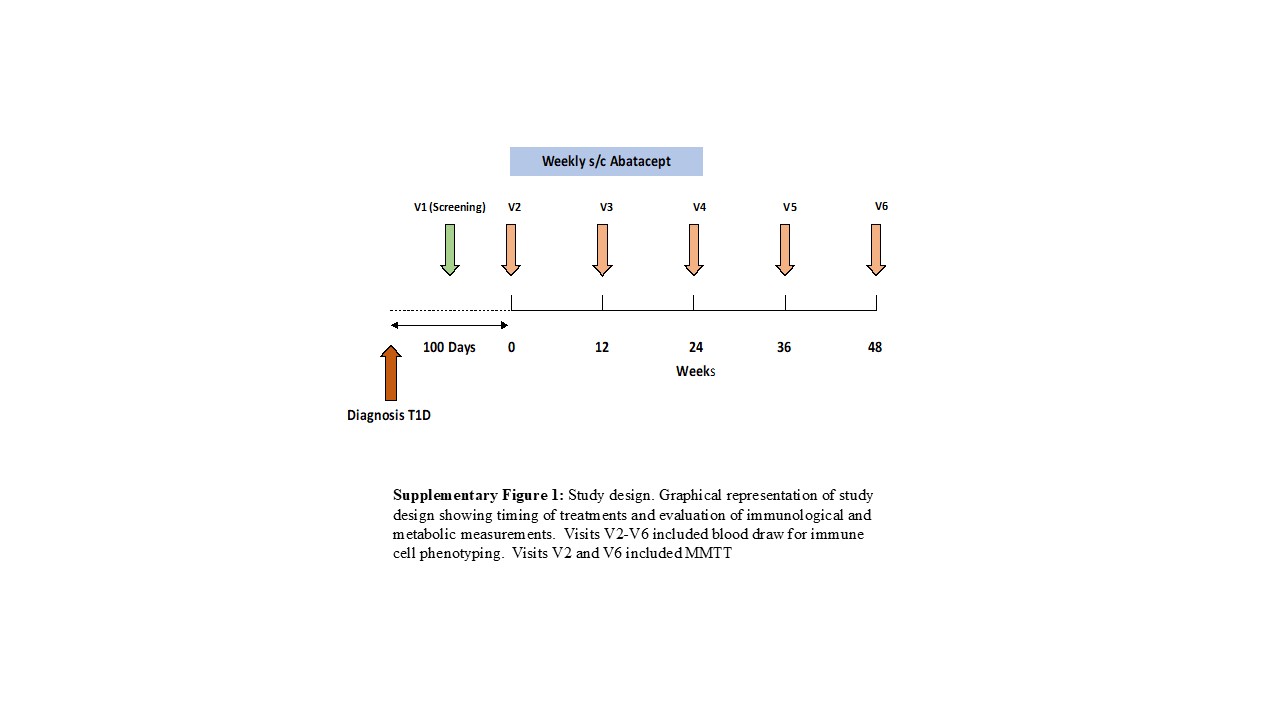

Supplement: Supplementary file 1 — Figure S1: Study design. Graphical representation of study design showing timing of treatments and evaluation of immunological and metabolic measurements. Visits V2‐V6 included blood draw for immune cell phenotyping. Visits V2 and V6 included MMTT. [file DMRR-41-e70074-s002.JPG]

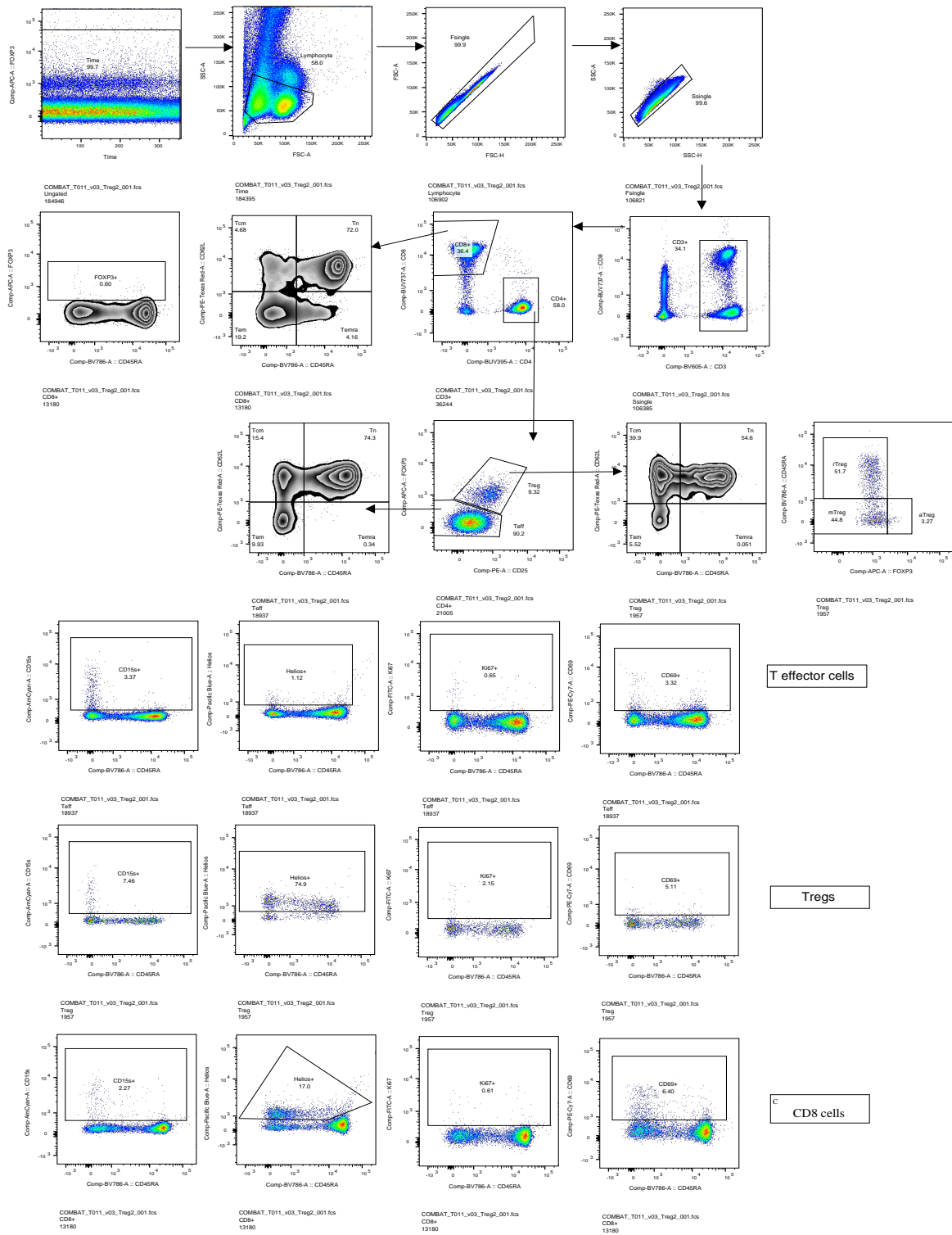

Supplement: Supplementary file 2 — Figure S2: Example flow cytometry gating strategy (Treg panel). [file DMRR-41-e70074-s001.pdf]
